# Fibrillatory Wave Amplitude: A Marker of Atrial Remodeling Rather Than a Chronological Clock?

**DOI:** 10.1111/anec.70175

**Published:** 2026-03-13

**Authors:** Emre Şener, Bektaş Murat, Bülent Görenek

**Affiliations:** ^1^ Department of Cardiology Eskişehir City Hospital Eskişehir Turkey; ^2^ Faculty of Medicine, Departmen of Cardiology Eskişehir Osmangazi University Eskişehir Turkey

Dear Editor,

We have meticulously reviewed the study by Li et al. (2026), which proposes surface electrocardiogram (ECG) fibrillatory wave (f‐wave) amplitude as a noninvasive surrogate for atrial fibrillation (AF) duration. While we commend the authors for their impressive sample size and the pragmatic pursuit of simple ECG markers, we believe that certain physiological and methodological paradoxes inherent in the study design warrant a more critical appraisal.

First, the core premise of the study using f‐wave amplitude to “date” AF assumes a linear temporal decline in voltage that may not account for the biological heterogeneity of atrial cardiomyopathy. Current evidence suggests that f‐wave amplitude is primarily a reflection of the degree of atrial fibrosis and low‐voltage zones rather than a simple chronological “clock” (Yin et al. 2017). By defining AF duration based on retrospective clinical records, which are notoriously prone to underestimating the silent burden of AF (Hindricks et al. 2021), the study risks a “circular validation.” In essence, the authors may be correlating an imprecise ECG marker with an equally imprecise clinical variable, potentially leading to overoptimistic conclusions regarding its predictive power.

Second, the reliance on lead V1 for manual amplitude assessment introduces significant confounding variables. Surface ECG amplitudes are highly sensitive to extracardiac factors, including chest wall impedance and electrode positioning. Without normalizing the f‐wave amplitude to the QRS complex or utilizing automated signal processing to minimize interobserver variability, the clinical generalizability of a few millivolts' difference remains questionable. Recent data in this Journal (Relander et al. 2024) emphasize the need for robust morphological analysis when linking f‐waves to clinical outcomes, suggesting that amplitude alone may be too simplistic a metric.

Third, the lack of formal diagnostic performance metrics, such as a Receiver Operating Characteristic (ROC) analysis to identify specific cutoff values for AF duration (e.g., > 10 years), limits the practical utility for the clinician. If the goal is to assist in the decision‐making process for catheter ablation, the study must move beyond statistical correlation and provide clinically actionable thresholds with defined sensitivity and specificity.

In conclusion, while the findings of Li et al. (2026) are hypothesis‐generating, the translation of these markers into clinical practice requires validation against objective measures of atrial substrate, such as delayed‐enhancement MRI or invasive electroanatomical mapping. We invite the authors to clarify how they accounted for the dominant influence of structural remodeling (Allessie et al. 2002) over purely temporal AF history.

## Author Contributions

Emre Şener and Bektaş Murat read the article and write the letter. Bülent Görenek contribute by his experience.

## Conflicts of Interest

The authors declare no conflicts of interest.

## Data Availability

The data that support the findings of this study are available from the corresponding author upon reasonable request.
